# Antiviral effects of interferon-stimulated genes in bats

**DOI:** 10.3389/fcimb.2023.1224532

**Published:** 2023-08-18

**Authors:** Dan Zhang, Aaron T. Irving

**Affiliations:** ^1^Zhejiang University-University of Edinburgh Institute, Haining, China; ^2^Second Affiliated Hospital, School of Medicine, Zhejiang University, Hangzhou, China; ^3^Centre for Infection, Immunity & Cancer, Zhejiang University-University of Edinburgh Institute, Haining, China; ^4^BIMET - Biomedical and Health Translational Research Centre of Zhejiang Province, China; ^5^College of Medicine and Veterinary Medicine, University of Edinburgh, Edinburgh, United Kingdom

**Keywords:** interferon pathway, bat, ISGs, IFN, antiviral

## Abstract

The interferon pathway is the first line of defense in viral infection in all mammals, and its induction stimulates broad expression of interferon-stimulated genes (ISGs). In mice and also humans, the antiviral function of ISGs has been extensively studied. As an important viral reservoir in nature, bats can coexist with a variety of pathogenic viruses without overt signs of disease, yet only limited data are available for the role of ISGs in bats. There are multiple species of bats and work has begun deciphering the differences and similarities between ISG function of human/mouse and different bat species. This review summarizes the current knowledge of conserved and bat-specific-ISGs and their known antiviral effector functions.

## 1 Interference by the interferon system

The innate immune system is an important natural barrier to infection and the first line of defense against virus invasion (Kawai and Akira, 2006; Carty et al., 2021). In most mammalian cells, pattern recognition receptors (PRRs) activate innate immunity by recognizing pathogen-associated molecular patterns (PAMPs) (Gürtler and Bowie, 2013). There are multiple classes of PRRs that are evolutionarily conserved in the majority of mammals, including Toll-like receptors (TLRs) (Lim and Staudt, 2013; Lester and Li, 2014), RIG-I like receptors (RLRs) (Kato et al., 2011), protein kinase R (PKR) (Kim et al., 2018), pyrin and hematopoietic interferon-inducible nuclear (PYHINs) domain family members (Bosso and Kirchhoff, 2020), cyclic GMP-AMP synthase (cGAS) (Hopfner and Hornung, 2020), and Nod-like receptors (NLRs) (Kim et al., 2016). PAMPs are conserved molecular features of foreign pathogens detected upon the infection of mammalian cells, with the most well-known being RNA or genomic DNA from viruses (Killip et al., 2015). PRRs bind to specific PAMPs of diverse pathogens: The endosomal TLRs 3/7/8, cytosolic RLRs, and PKR detect various forms of cellular or viral RNAs in all mammalian cells tested so far (Kato et al., 2011; Majer et al., 2017; Kim et al., 2018), while TLR9, PYHINs, STING, and cGAS act as sensors for DNA viruses (Majer et al., 2017; Bosso and Kirchhoff, 2020; Hopfner and Hornung, 2020). The lack of all PYHIN family members, across all bat genomes tested to date, seems to be the first glimpse of differential innate immunity in this reservoir host (Ahn et al., 2016).

Following the activation of PRRs, host cells activate multiple adapter molecules, eventually triggering interferon regulatory factors (IRFs) and NFкB to drive secretion of cytokines, such as interferons (IFN), that suppress early viral infection (Grandvaux et al., 2002; Negishi et al., 2018). IFNs are divided into three main subtypes with Type I and III most commonly associated with initiating viral clearance, through both autocrine or paracrine signaling loops (Onoguchi et al., 2007). Once IFNs bind their corresponding receptors, the JAK-STAT signaling pathway is activated. Phosphorylation of JAKs/Tyk2, among others, activates STATs to form homo- or hetero-dimers that translocate to the nucleus to bind specific ISRE promoter sites in interferon-stimulated genes (ISGs) and activate transcription of over 1,000 ISGs, depending on the cell type (Schneider et al., 2014; Schreiber and Piehler, 2015).

Although the function of several hundred of these ISGs has been analyzed, some for over 30 years, the antiviral efficacy of many ISGs, albeit their most important function in this cascade, has been poorly characterized (de Veer et al., 2001). IFNs, via activation of ISGs, are known to activate both infected and the surrounding uninfected cells, to induce an antiviral state and mediate immune cell defense against viral infection (Schoggins and Rice, 2011). Many antiviral ISGs have been characterized in detail, yet there are many more ISGs in this vast gene pool requiring further detailed studies to understand their true relevance in the context of infection.

## 2 Antiviral pathways in bats

### 2.1 The unique interferon response?

Bats, the only mammals with the ability of powered flight, have a global distribution of more than 1,462 species; Simmons and Cirranello, 2023). Since the severe acute respiratory syndrome coronavirus (SARS-CoV) outbreak of 2003, researchers began to focus more on bats as reservoir hosts for viruses due to the identification of a SARS-like virus in bats (Lau et al., 2005) along with multiple similar coronavirus genes that could recombine to build SARS-CoV (Hu et al., 2017). The current COVID-19 pandemic has brought further attention to bats as reservoirs of zoonotic viruses (Irving et al., 2021) and further highlighted some key features of their immune systems. Bats that are clinically healthy appear to harbor many viruses that may be fatal to humans, such as ebolaviruses (Leroy et al., 2005; Hayman et al., 2010), lyssaviruses (Wang et al., 2005; He et al., 2014), Marburg virus (Towner et al., 2007; Towner et al., 2009), Nipah virus (Rizzo et al., 2017; Darcissac et al., 2021; Zhu et al., 2022), and coronaviruses (Ge et al., 2013; Hu et al., 2017; Zhou et al., 2021). Yet, despite this, bats seem to have evolved the ability to clear viral infections asymptomatically (Irving et al., 2021). Although antiviral innate immunity is relatively conserved among mammals, bats have some distinctive immune features that hint at the ability to co-exist with viruses (Clayton and Munir, 2020). One recent study used computer modeling of the IFN antiviral pathways in bats to suggest that their unique immune system may have driven the evolution of viruses to the detriment of humans and other animals (Brook et al., 2020).

While the majority of PRRs are evolutionarily conserved in bats, previous studies have suggested that some homologs of human viral PRRs appear to have undergone evolutionary selection pressure (Escalera-Zamudio et al., 2015; Jiang et al., 2017). The full-length mRNA transcripts of TLR1–TLR10 were nevertheless sequenced in one bat, the black flying fox (*Pteropus alecto)*, where an almost complete TLR13 pseudogene was detected, a gene lacking in humans (Cowled et al., 2011).

Constitutive expression of IFN-α protein was found in four bat species, unlike its exclusive inducibility in humans, and this basal expression level of IFN-α in bats was not associated with disease, suggesting a unique IFN response in bats (Bondet et al., 2021). One study showed that *P. alecto* has fewer IFN genes compared to human, with only three IFN-α genes, revealing an unusual basal expression pattern not described in other mammals (Zhou et al., 2016). This suggests that some bats have a high basal expression level of this contracted IFN family and may affect viral infection. Another study in a closely related fruit bat (*Rousettus aegyptiacus*) showed expansion of a type I IFN locus with a large number of IFN-ω detected and no baseline IFN-α expression detected (Pavlovich et al., 2018). This highlights species-specific differences in the bat IFN systems that may lead to differential activation of ISGs. Additionally, IFN-κ and IFN-ω were cloned from a European serotine bat (*Eptesicus serotinus*) and functional studies showed that its IFN-κ gene resided outside of the typical type I IFN locus, indicating independent genetic development in bat species (He et al., 2014). While most immune gene sequences are largely conserved, IFN-κ and IFN-ω subtypes are diversified among different bat families, which is suggestive of evolutionary adaptation of IFNs within each bat species (He et al., 2014). Of note, both IFN-κ and IFN-ω inhibited the replication of lyssaviruses in *E. serotinus* bat brain cells, whereas IFN-α and IFN-β were strongly associated with lyssavirus infection in human and mouse models (Wang et al., 2005; He et al., 2014). In contrast to the contractive IFN-α genes, a sequencing analysis of IFN genes in large flying fox bat (*Pteropus vampyrus*) and little brown bat (*Myotis lucifugus*) showed that both bat species have significantly expanded IFN-ω and IFN-δ genes compared to their human counterparts (Kepler et al., 2010) matched to the similar expansion later observed in *R. aegyptiacus (* Nikaido et al., 2020).

Type I IFN expression is induced by a variety of interferon regulatory factors (IRFs) (Miyamoto et al., 1988), among which IRF3 and IRF7 are most significant (Ning et al., 2011; Al Hamrashdi and Brady, 2022). Previous functional studies in bats have shown that IRF3 is capable for inducing antiviral signaling after poly(I:C) stimulation and Middle East Respiratory Syndrome Coronavirus (MERS-CoV) treatment in big brown bat (*Eptesicus fuscus*) kidney cells (Banerjee et al., 2019). *E. fuscus* IRF3 nucleotide sequences are also clustered separately from human and non-human primate IRF3 sequences, suggesting some degree of gene divergence in bats (Banerjee et al., 2019). Further research on 11 species of bat IRF3s emphasized positive selection at the S185 residue, and substituting a human-like Leucine residue for Serine reduced the antiviral protection of bat IRF3 (Banerjee et al., 2020a). Additional work showed that bat IRF1, 3, and 7 are elevated in most bat tissues of *E. spelaea* and *P. alecto*, resulting in a faster ISG response to virus infection (Zhou et al., 2014; Irving et al., 2020). IRF1, an ISG itself, is upregulated during infection and has shown to have antiviral efficacy against MARV/EBOV, IAV, PRV3M, and MERS-CoV (though only limited comparisons to human IRF1 are available) (Irving et al., 2020; Kuzmin et al., 2021).

In human, type I IFNs bind to IFN-α receptors (IFNAR1/2) to activate downstream pathways regulating ISGs (Lamken et al., 2004). In *P. alecto* and consequently other species, signal recognition of bat or universal IFN-α was confirmed to be dependent on IFNAR2, in conjunction with IFNAR1 (Zhang et al., 2017), and type III IFNs through IFNAR1, in conjunction with IL10R2, just like human (Zhou et al., 2011). Short-tailed bat (*Carollia perspicillata*) kidney cells from the *Phyllostomidae* family are unresponsive to human IFNα treatment, similar to *P. alecto*, suggesting species specificity of their IFN-α receptors (Fuchs et al., 2017). The majority of bat cells, however, have proven capable of stimulation via the universal IFN-α (Shaw et al., 2017). The antiviral ISG immune response appears largely consistent across mammals (Shaw et al., 2017; McDougal et al., 2022). However, bat cells seem to be able to limit cellular inflammation, while maintaining this antiviral IFN response. When human and *E. fuscus* kidney cells were stimulated with Poly(I:C), only human cells expressed significant amounts of tumor necrosis factor α (TNFα), a pro-inflammatory signaling cytokine. A potential repressor binding motif (c-Rel) was identified in *E. fuscus* that limits the levels of bat TNFα transcripts and thus inhibits inflammatory pathologies (Banerjee et al., 2017). Other observations to match this include the absence of PYHIN proteins (Ahn et al., 2016), sometimes linked to IFN cascade (Xie et al., 2018), and suppression of a central inflammasome sensor, NLR family pyrin domain containing 3 (NLRP3), via a novel splice variant and alterations in the leucine-rich repeat domain leading to suppressed function of NLRP3 in addition to repressed transcription (Ahn et al., 2019).

Although bat cells produce IFNs for their canonical antiviral response, the expression of cytokines involved in inflammation is limited. In humans, the activation of inflammasomes leads to strong pathological responses that cause severe tissue damage (Karki et al., 2017). Activation of interleukin 1β (IL-1β) processing via caspase is suppressed in bats (Shaw et al., 2017; Goh et al., 2020) and unlike the intense and chronic inflammatory response of other mammals, bats can partially suppress the immune response against viruses (David et al., 2022). Other cytokines such as IL-10 and transforming growth factor β (TGFβ), with a potential anti-inflammatory function, appear to be upregulated in bats during *in vivo* infection with viruses (Burke et al., 2023). Compared to mouse (*Mus musculus*) macrophages, the greater mouse-eared bat (*Myotis myotis*) macrophages possessed a robust anti-inflammatory response that is most likely related to viral tolerance in bats (Kacprzyk et al., 2017).

Even in the absence of stimulation, ISG expression levels in bat cells were generally higher than in matched human cells (Zhou et al., 2016; Shaw et al., 2017). Additional immunological control may be due to reduced IFN production, post-stimulation, or rapid turnoff (Schountz et al., 2017; Irving et al., 2020). The expression of ISGs in human kidney cell lines increased for long periods of time after treatment with type I IFN-α, whereas the expression of ISGs in matched bat cells generally increased rapidly and then decreased after treatment with IFN-α (De La Cruz-Rivera et al., 2018).This increased basal level of ISG expression in bats may enable the bat immune system to respond more quickly to invading pathogens and allow faster clearance of virions with less damage to cells. While the majority of the immune system seems conserved across mammals, these bat-specific evolutionary events have led to some bat-specific immune characteristics, giving bats a powerful innate antiviral response. Further studies of virus–host interactions in bats may allow researchers to understand cross-mammal antiviral evolution and allow an application of the findings for modulation of human health.

### 2.2 RNA virus sensing

The evolutionary conserved RNA recognition PRRs suggest similar pathways leading to ISG induction (Zhang Y, et al, 2013; Tarigan et al., 2020). Immortalized bat kidney cells with knockdown of TLR3, RIG-I, and MDA5 decreased IFN-β expression and led to increased viral replication following infection with RNA viruses, highlighting their importance for antiviral gene induction in bats, as is similarly observed in humans (Tarigan et al., 2021). In bats, 63% of TLR8 genes have evolved under purifying selection, and 7% of TLR8 genes have sustained selection at specific loci (Jiang et al., 2017). The TLR8 gene in bats shows a wide range of sequence variation compared with other mammals, which may result in differential ability to recognize PAMPs in different bat species (Schad and Voigt, 2016; Jiang et al., 2017).

Structural and tissue specificity studies of RIG-I and MDA5 receptors in bats largely suggest similarities with humans and other mammals (Yoneyama et al., 2005; Cowled et al., 2012; Wang et al., 2022). Detection of synthetic dsRNA poly(I:C) in *P. alecto* kidney cells resulted in the elevation of IFNβ and expression of these two PRRs (Cowled et al., 2012). Another study on the common vampire bat (*Desmodus rotundus*) found that RIG-I and MDA5 were both early inducible genes after poly(I:C) induction (Sarkis et al., 2018a), suggesting that they may respond more quickly to viral infections. Duplication and diversification of PKR among bats also suggest evolutionary changes linked to RNA sensing in bats (Jacquet et al., 2022). It was recently identified that the amino acid sequence of the Brazilian free-tailed bat (*Tadarida brasiliensis*) MDA5 is less conserved among different species and is more evolutionarily similar to the homolog of human (Wang et al., 2022). A similar mRNA upregulation of MDA5 is detected in response to infection in bats, indicating a conserved MDA5 pathway for activating innate immunity against RNA viruses (Wang et al., 2022). Combined, this suggests that differential induction patterns of some bat ISGs, while maintaining a similar functional mechanism, occurs for some proteins while induction of other bat ISGs is largely conserved. As these pathways are fundamental sensing pathways required to sense both cellular DAMPs and foreign PAMP signals, for a variety of pathogens, whether a slightly altered PRR function and consequent gene induction have an impact on antiviral immunity is yet to be fully elucidated.

### 2.3 DNA virus sensing

Viral detection of PRRs is a complicated process involving endosomal TLR9, cGAS, and PYHIN family members to identify unusual self- or foreign DNA (Briard et al., 2020). While TLR9 recognizes unmethylated CpG DNA and interacts with myeloid differentiation primary response 88 (MyD88) to initiate the production of type I IFN, others expand the specificity and effectiveness of the immune response to viral DNA (Saber et al., 2022). TLR9 has higher expression in bats than other mammals and additionally undergoes positive selection during evolution (Escalera-Zamudio et al., 2015). In most human cells, the cyclic GMP-AMP receptor stimulator of interferon genes (cGAS-STING) signaling pathway detects the cytoplasmic DNA from pathogens to upregulate ISGs and drive inflammation (Hopfner and Hornung, 2020). Loss of S358 in bat STING results in a diminished ability to induce interferon and therefore ISGs (Xie et al., 2018). High-speed flight in bats and flight-induced hyperthermia may potentially cause more cellular damage in bats, producing an increase in cytosolic DNA (Hock, 1951; Banerjee et al., 2020b). Additionally, bats have undergone evolutionary adaptations in DNA sensing and repair pathways, presumably in adaptation to flight.

Genomic analysis of the PYHIN gene family, across 10 bat species, showed complete loss of this gene family in bats (Ahn et al., 2016). All other mammals have at least one gene member of the PYHIN family, with only bats having lost the entire family of genes (Ahn et al., 2016). This absence of the PYHIN family in bats, still observed with newly released genomes, limits the activation of inflammatory responses from DNA damage, in addition to minimizing ISG induction from cytosolic DNA, as observed in other species via IFI16/IFI207 and related family members (Xie et al., 2018; Baran et al., 2023). A large-scale genomic screen of bats further confirmed that these genes are deleted in bats (Moreno Santillán et al., 2021).

Previous analyses suggest that the majority of positively selected genes in bats are associated with innate immunity and DNA damage (Hawkins et al., 2019). Dampened and altered DNA sensing may be an adaptation to this damaged self-DNA, yet the impact of altering these sensing pathways on ISG induction is yet to be fully understood.

## 3 ISGs in bats

### 3.1 Functions of ISGs in bats

While the majority of observed PRR responses in bats are largely matched to those in other mammals, a number of downstream effector genes appear to be altered, potentially modifying the antiviral mechanism of innate immunity (Schoggins, 2018). Through utilizing a matched cross-species platform comparison, researchers concluded that each species (not just bats) has both a unique repertoire of species-specific-ISGs and also a conserved “core” of ISGs common to certain phylogenies or across all mammals (Shaw et al., 2017). In humans, hundreds of ISGs have been identified, yet the number in each bat species remains undetermined. Transcriptome analysis of *P. alecto* kidney cells post-induction revealed a considerable number of ISGs with unknown function and also genes previously unknown to be ISGs in terms of their IFN inducibility (Zhang et al., 2017). This suggests a complex regulatory mechanism that differs from our understanding of humans or mice.

The recent COVID-19 pandemic has reawakened interest in cross-species comparisons of ISG function as further information is required for the role of ISGs in direct antiviral effector function, autoimmunity suppression, restriction of inflammation, and even tumor suppression. An example in one species shows that many immune-related genes in *R. aegyptiacus* have undergone positive selection compared to humans, including MX1, OAS1, and ISG15 (Pavlovich et al., 2018). Similar trends have been observed for whole branches of the bat phylogenetic tree (Tian et al., 2023).

As important hosts of zoonotic viruses, the innate immunity of bats has gained much interest, and a number of bat ISGs are enriched in pathways involving cancer, suggesting additional roles in tumorigenesis suppression (Zhang et al., 2017) with bats considered to have a low incidence of tumors in wild individuals (Brook and Dobson, 2015). ISGs are involved in a range of cellular functions, but of importance to viral spillover into human populations, the antiviral function is arguably the most important. While previous work covers multiple aspects of the bat IFN system and how it is activated and expressed (Banerjee et al., 2020b), herein we summarize the antiviral effector role of bat ISGs.

### 3.2 Functionally conserved ISGs

Previous studies have identified a diverse cohort of ISGs responsive to different viruses in various human cells through overexpression screening (Schoggins et al., 2011). There is believed to be a strong evolutionary conservation of innate immunity in vertebrates, with the genetic identification of multiple ISGs previously identified in human or mice. In addition to the essential effector TLR molecules, cGAS, RIG-I, MDA5, MYD88, IFNAR1/2, STAT1/2, and the ISGs related to interferon induction IRF1/3/7/9 are present in all bat species. Multiple studies identify ISGs that are upregulated in response to IFNs or viruses in various bat cells and *in vivo (* Zhang et al., 2017) in bats, though there are only limited studies directly examining antiviral efficacy (Guito et al., 2021). Some conserved ISGs with known antiviral function, such as ISG15/20, Mx1/2, OAS1, ADAR1, and PKR, are present in all bats and often induced upon infection (Papenfuss et al., 2012; Shaw et al., 2017; Sarkis et al., 2018b). Some appear to be undergoing evolutionary selection pressure and others have undergone gene expansion in bats (as well as other mammals) (**Table 1**). In *P. alecto*, the three antiviral ISGs, Mx1, OAS1, and PKR, are highly conserved structurally and functionally, with Mx1 and OAS1 induced in a highly IFN dose-dependent manner (Zhou et al., 2013). Bat Mx1 is the functional homolog of human MxA and has a similar antiviral capacity against IAV, VSV, La Crosse Virus, and H18N11 IAV. Ectopic expression of bat Mx1 protein from three different families in human 293T cells significantly reduced the activity of viral polymerase, suggesting a conserved and similar function to human (Fuchs et al., 2017; Ciminski et al., 2019; Hölzer et al., 2019; McKellar et al., 2023), though compared with humans, bat Mx1 proteins have undergone positive selection at both the N and C terminus (Fuchs et al., 2017). There does appear to be species-level specificity in antiviral effector function across six bat species, however, when profiled against certain IAV subtypes and RVFV (Fuchs et al., 2017). The human OAS1 gene promoter has one typical IFN-stimulated response element (ISRE), whereas the bat counterpart has two, suggesting a conserved function yet altered regulation across phylogenies (Zhou et al., 2013). Indeed, in *D. rotundus* bats, the OAS1 gene has two orthologs, with differential expression yet similar antiviral function (Sarkis et al., 2018b). OAS3 from *R. aegyptiacus* also exhibits antiviral efficacy against Sindbis virus (SINV) and Vaccinia virus (VACV), suggesting a similar conservation in antiviral function (Li et al., 2019). EIF2AK2 (PKR) shows a conserved functional inhibition in *D. rotundus* bats, but shows poor expression in several bat species (Sarkis et al., 2018b; Jacquet et al., 2022). ADAR1, a well-known RNA editing antiviral protein (George and Samuel, 1999), has similar antiviral function against endosomal viruses to human, yet seems to exhibit and enhanced ADAR function in bat lung and kidney cells against EBOV infection (Sarkis et al., 2018b; Whitfield et al., 2020). The amino acid sequence of ADAR1 was found to have high homology across mammals, yet some core sequences were conserved in bats (Sarkis et al., 2018b). MTHFD1 is conserved antiviral effector gene showing antiviral efficacy in humans and *P. alecto* against Mumps virus and IAV (Anderson et al., 2021). Another functionally conserved antiviral effector in bats is IFITM3. Studies in microbat (*Myotis lucifigus*) suggest that while bat IFITM3 has undergone adaptive evolution, it remains largely similar in its antiviral capacity against a range of endosomal viruses (Benfield et al., 2015; Benfield et al., 2020).

**Table 1:** Antiviral effector ISGs in bats.

ISG	Species	Experimental models	Notes	Ref.
IRF1	*P. alecto*; *R. aegyptiacus*	Immortalized PakiT03 cell line (kidney); Immortalized R06E7 cell line (fetus)	Antiviral against MARV/EBOV, antiviral against IAV, PRV3M, MERS-CoV	(Irving et al., 2020; Kuzmin et al., 2021)
IRF3/7	*P. alecto*	Immortalized PakiT03 cell line (kidney), validation in VeroE6/A549	Antiviral against HSV1, PRV3M, IAV, MERS-CoV	(Irving et al., 2020)
DDX58 (RIG-I)	*P. alecto;* *R. aegyptiacus;* *R. ferrumequinum*	RNA/cDNA from *P. alecto* peripheral blood mononuclear cells (blood); Immortalized R06E7 cell line (fetus); Established BKT1 cell line (kidney), overexpression Hek293	Antiviral against MARV/EBOV, conserved antiviral function against EMCV and JEV	(Cowled et al., 2012; Tarigan et al., 2020; Kuzmin et al., 2021)
BST2 (Tetherin)	*E. buettikoferi;* *H. monstrosus;* *M. daubentoniid; M. macropus;* *P. alecto*	Established EpoNi/22.1 cell line (kidney); Established HypNi/1.1 cell line (kidney); Established MyDauNi/2c cell line (kidney); RNA/cDNA from *M. macropus* (spleen); RNA/cDNA from *P. alecto* (spleen)	Multiple BST2 paralogs. Inhibits HIV-1/EBOV release, NiV replication (but not EBOV replication). Inhibition of EBOV-GP more potent in fruit bats. Different isoforms exhibit enhanced restriction of MARV.	(Hayward et al., 2018; Hölzer et al., 2019; Hoffmann et al., 2019; Nehls et al., 2019)
IFIH1 (MDA5)	*R. aegyptiacus;* *R. ferrumequinum;* *T. brasiliensis*	Immortalized R06E7 cell line (fetus); Established BKT1 cell line (kidney); Established TB1Lu cell line (lung)	Antiviral against MARV/EBOV, conserved MDA5 function against VSV, EMCV, and JEV	(Tarigan et al., 2020; Kuzmin et al., 2021; Wang et al., 2022)
IFIT1	*R. aegyptiacus*	Immortalized R06E7 cell line (fetus)	Antiviral efficacy against EBOV and MARV	(Kuzmin et al., 2021)
IFITM3	*M. myotis*	Primary fibroblasts and lung cells from *M. myotis*	Conserved antiviral function yet positively selected in certain species.	(Benfield et al., 2015; Benfield et al., 2020)
ADAR	*D. rotundus;* *E. buettikoferi*	Established FluDero cell line (lung); Established EpoNi/22.1 cell line (kidney), validated 293T	Normal antiviral function against endosomal viruses, enhanced ADAR function in bat cells against EBOV	(Sarkis et al., 2018b; Whitfield et al., 2020)
APOBECs	*P. alecto*	RNA/cDNA from *P. alecto* (spleen, lymph node, white blood cells, bone marrow and thymus), validation in Hek293T	Differential efficacy of APOBEC3 orthologs against retroviruses expanded gene family in bats	(Hayward et al., 2018; Moreno Santillán et al., 2021)
MORC3	*M. daubentoniid;* *P. alecto;* *P. vampyrus*	Established MyDauNi/2c cell line (kidney); Immortalized PaKiT03 cell line (kidney); Primary PPVK cells and Immortalized PVK4 cells (kidney)	An ISG in bats, but not in human, has an antiviral function against HSV1, CMV	(Glennon et al., 2015; Zhang et al., 2017; Hölzer et al., 2019)
MX1	*C. perspicillata;* *H. monstrosus;* *M. daubentoniid;* *P. pipistrellus;* *R. aegyptiacus;* *S. lilium;*	Established CarLu/1 cell line (kidney); Established HypNi/1 cell line (fetal kidney); Established MyDauNi/2c cell line (lung); Established PipNi/1 cell line (kidney); Established RoNi/7 cell line (kidney); RNA/cDNA from S.lilum	Multiple MX paralogs, conserved function against IAV, VSV, and La Crosse virus, and restricts H18N11 bat-IAV. Species-specific IAV/RVFV restriction efficiency.	(Fuchs et al., 2017; Ciminski et al., 2019; Hölzer et al., 2019; McKellar et al., 2023)
MX2	*P. alecto*	Established PakiT01/PabrH02/PafesV40T/PaluT02 cell lines (kidney/brain/fetus/lung)	*P. vampyrus* fails to restrict HIV, unlike human and *P. alecto*-restricted HIV	(Morrison et al., 2020; Ohkura et al., 2023)
OAS1a/b	*D. rotundus*	Established FluDero cell line (lung)	Normal antiviral function of both isoforms but differential expression	(Sarkis et al., 2018b)
MTHFD1	*P. alecto*	Genome of Paki cells (kidney)	Conserved antiviral function against mumps and IAV.	(Anderson et al., 2021)
OAS3	*R. aegyptiacus*	Established RoNi/7 cell line (kidney)	OAS3 more potent than OAS1/2 against Sindbis and Vaccinia.	(Li et al., 2019)
PKR(EIF2AK2)	*D. rotundus, M. myotis*	Established FluDero cell line (lung); Tissues from *Myotis* species, including *M. myotis*, *M. velifer*, *M. riparius*, *M. nigricans*, *M. mystacinus*, *M. emarginatus*, and *M. bechsteinii*	Normal antiviral function in *D. rotundus*. Gene expansion of PKR in myotis bats to evade viral antagonism. Differential function of PKR1/2 against VACV and VSV and evasion against Herpes viruses.	(Sarkis et al., 2018b; Jacquet et al., 2022)
Trim5α	*P. alecto;* *P. dasymallus;* *R. leschenaultia*; *E. fuscus*; *E. helvum*; *M. schreibersii*; *M. fuliginosus*; *T. brasiliensis*; *E. nilssonii*; *R. aegyptiacus*; *E. crypturus*	Established PakiT01/PabrH02/PafesV40T/PaluT02 cell lines (kidney/brain/fetus/lung); Primary FBKT-1 *Pteropus dasymallus* (kidney); Established DemKT-1 *Rousettus leschenaultia* (kidney); R06E *R. aegyptiacus*; ZFBK11-97 *Epomophorus crypturus*; ZFBK13-76E *Eidolon helvum*; Immortalized BKT-1 *Rhinolophus ferrumequinum* (kidney); YubKT-1 *Miniopterus fuliginosus* (kidney); YubKT-2 *M. fuliginosus* (kidney); SuBK12-08 *M. schreibersii*; Established Tb1.Lu *Tadarida brasiliensis* (Lung); Immortalized EfK3B *Eptesicus fuscus* (kidney); EnK E. nilssonii (kidney); Validated in HeLa.	Restricts MLV but not HIV, unlike human.	(Morrison et al., 2020; Ohkura et al., 2023)
USP18	*P. alecto;* *R. aegyptiacus*	Immortalized PakiT03 cell line (kidney); Spleen RNA/cDNA of *P. alecto;* 2nd/3rd gen captive-bred *R. aegyptiacus* bats (Uganda origin)	A negative regulator of IFN pathways or positive regulator of MAVS, highly induced upon MARV or PRV3M infection.	(Irving et al., 2020; Guito et al., 2021; Hou et al., 2021)
RNASEL	*P. alecto*	Immortalized PaBr/PaLu/PaKi cell lines (brain/lung/kidney)	Antiviral effector of the 2′-5′-OAS pathway, RNASEL, highly IFN-inducible in bats but not in humans. Highly induced with SUDV	(Zhang et al., 2017; De La Cruz-Rivera et al., 2018)
RTP4	*P. alecto*	Immortalized PakiT03 cell line (kidney), overexpressed Huh7.5 cells.	Antiviral effector against flavivirus—ZikaV, YFV, DENV (similar to human), and HCV, EAV, and hCoV	(Boys et al., 2020)
ISG15	*M. davidii*	Protein work	Molecular analysis suggests similar function and activation though potentially different binding affinity.	(Langley et al., 2019)

Genes indicated in blue are found to be expressed higher basally than human in a matched cell line*. Genes in orange are not ISGs in humans but are in bats (and some other mammals). *A detailed multi-cell line/multi-bat species comparison has not been conducted.

The RLR pathway seems intact among bats with IFIH1 (MDA5) and DDX58 (RIG-I) having antiviral activity against MARV/EBOV, similar to humans, and a conserved antiviral function against EMCV and JEV for RIG-I (Cowled et al., 2012; Tarigan et al., 2020; Kuzmin et al., 2021; Wang et al., 2022). These genes are often highly expressed in bats and enriched upon infection, suggesting the importance of these pathways in controlling infection.

While bats may exhibit a range of conserved antiviral functions to humans, multiple bat ISGs are undergoing adaptive evolution, suggesting possible changes to their function (Zhang G, et al., 2013). Some of those with seemingly similar function to human are also under selection pressure, indicating that yet other pathogens may trigger differential responses when compared to humans. Other bat ISGs do indeed show differential function when compared directly to human or mouse. In addition, several bat genes are shown to be bat-specific ISGs, or at least ISGs in other mammals and not in humans, indicating the importance of this innate response in controlling infection. The identification of which ISGs under positive selection may directly play a role in viral susceptibility and zoonosis can assist in predicting both targets and possible control of novel spillover events.

### 3.3 Atypical ISGs

A meta-analysis across 18-bat genomes suggests that a large number ISGs in bats are undergoing positive selection (Hawkins et al., 2019). Although ISGs and immune genes in general are more likely to be undergoing selection, this proportion is even higher in bats compared to other mammals. Given there are 1,462 species of bats (Simmons and Cirranello, 2023), each potentially with species-specific ISG function, there is a great potential for atypical ISGs with an inextricably powerful antiviral immune system (Glennon et al., 2015; De La Cruz-Rivera et al., 2018). Approximately 200 to 300 genes were upregulated in NDV-infected primary and immortalized *P. vampyrus* kidney cells, with some genes typical of antiviral pathways, including RIG-I, MDA5, IRF1, and ISG15. Yet, there are also genes not previously identified as ISGs, including RND1, SERTAD1, CHAC1, and MORC3 (Glennon et al., 2015). MORC3 transcripts were significantly increased after treatment with IFNα and NDV, though not observed in human A549 cells (Glennon et al., 2015). The Interferon database recognizes MORC3 as only a weak ISG in both mice and human (Rusinova et al., 2013).This was later confirmed in several other species (Zhang et al., 2017; De La Cruz-Rivera et al., 2018; Hölzer et al., 2019). Recent studies have begun characterizing the antiviral potential of MORC3 (Zhang et al., 2019; Gaidt et al., 2021). A similar bat-specific ISG, confirmed in several species, is RNASEL. While its antiviral effector function is well characterized, it is not an ISG itself in humans, suggesting a quicker activation of the OAS pathway in bats compared to humans (Zhang et al., 2017; De La Cruz-Rivera et al., 2018; Li et al., 2019).

Tetherin (BST2) encodes a tethering protein that inhibits the release of several enveloped viruses from infected cells (Neil, 2013; Hayward, 2022). BST2 is induced by IFN in various fruit bat cells and significantly inhibits the release of Ebola-like virions and also HIV, though with slight differences compared to human BST2 (Neil et al., 2007; Hoffmann et al., 2019). The inhibition of EBOV-GP was also more potent in fruit bats compared to humans. Similarly, different paralogs of BST2 exhibited differential restriction efficacy against MARV and in inhibiting the replication of Nipah virus (Hayward et al., 2018; Hölzer et al., 2019; Hoffmann et al., 2019; Nehls et al., 2019).

Unlike the relatively conserved MX1, MX2 exhibited differential behavior towards HIV-1, possibly being influenced by differential exposure to pathogens between bats and humans (Fuchs et al., 2017; Morrison et al., 2020). Interestingly, *P. vampyrus* MX2 failed to restrict HIV (unlike human), yet a closely related bat species, *P. alecto*, has the capacity to inhibit HIV (Morrison et al., 2020; Ohkura et al., 2023). Trim5α of *P. vampyrus* and *P. alecto* can restrict MLV, but not HIV, in contrast to the behavior of human, again suggesting bat species-specific adaptations to different retroviruses (Morrison et al., 2020; Ohkura et al., 2023). APOBCE3 is an antiviral restriction factor that inhibits normal replication of retroviruses (Chen et al., 2006). A variety of APOBEC3 paralogs have been identified in bats, and their subtypes can limit the infectivity of HIV, proving a strong and unique antiviral function (Hayward et al., 2018). Phylogenetic amplification of APOBEC3 and gene families was also found in a large-scale genome study of 37 bat species, indicating enhanced evolution of this gene family (Moreno Santillán et al., 2021).

A detailed study attempting to examine why *R. aegyptiacus* fruit bats have minimal effects upon exposure to virus utilized 23 ISGs cloned to test their antiviral effects against Ebola virus and Marburg virus. The results show that overexpression of RIG-I, IFIT1 (ISG56), and IRF1 significantly inhibited viral replication, though detailed comparisons of their human counterpart have not been studied in this context (Kuzmin et al., 2021). A conserved negative regulator of the IFN pathway, USP18, is highly induced in bats and has a dual-feedback role in being a positive regulator of the MAVS pathway for viral sensing. This gene is highly induced upon MARV or PRV3M infection in bats and bat cells, suggesting perhaps direct antiviral function, while inhibiting systemic IFN secretion (Hou et al., 2021). A newly discovered antiviral ISG, RTP4, shows conserved function to human against ZikaV, YFV, and DENV, yet exhibits enhanced function in bats against HCV, EAV, and hCoV, compared to humans (Boys et al., 2020). In certain families of bats, EIF2AK2 (PKR) is expanded with multiple paralogs of the gene exhibiting differential expression and viral-specific function against VACV and VSV. This suggests that gene duplications were followed by evolution against specific pathogens, to allow differential viral specificity, in a manner not observed in other mammals. The evolution of these genes appears to be linked to evasion of viral proteins targeting them (Sarkis et al., 2018b; Jacquet et al., 2022). Additionally, overexpression of IFN-ω in *R. aegyptiacus* kidney cells significantly inhibits Marburg virus replication, suggesting unique functions from these IFN-subtypes via the ISGs they induce, including those ISGs with bat-specific differences in function (Pavlovich et al., 2020).

## 4 Conclusion

**Figure 1:**
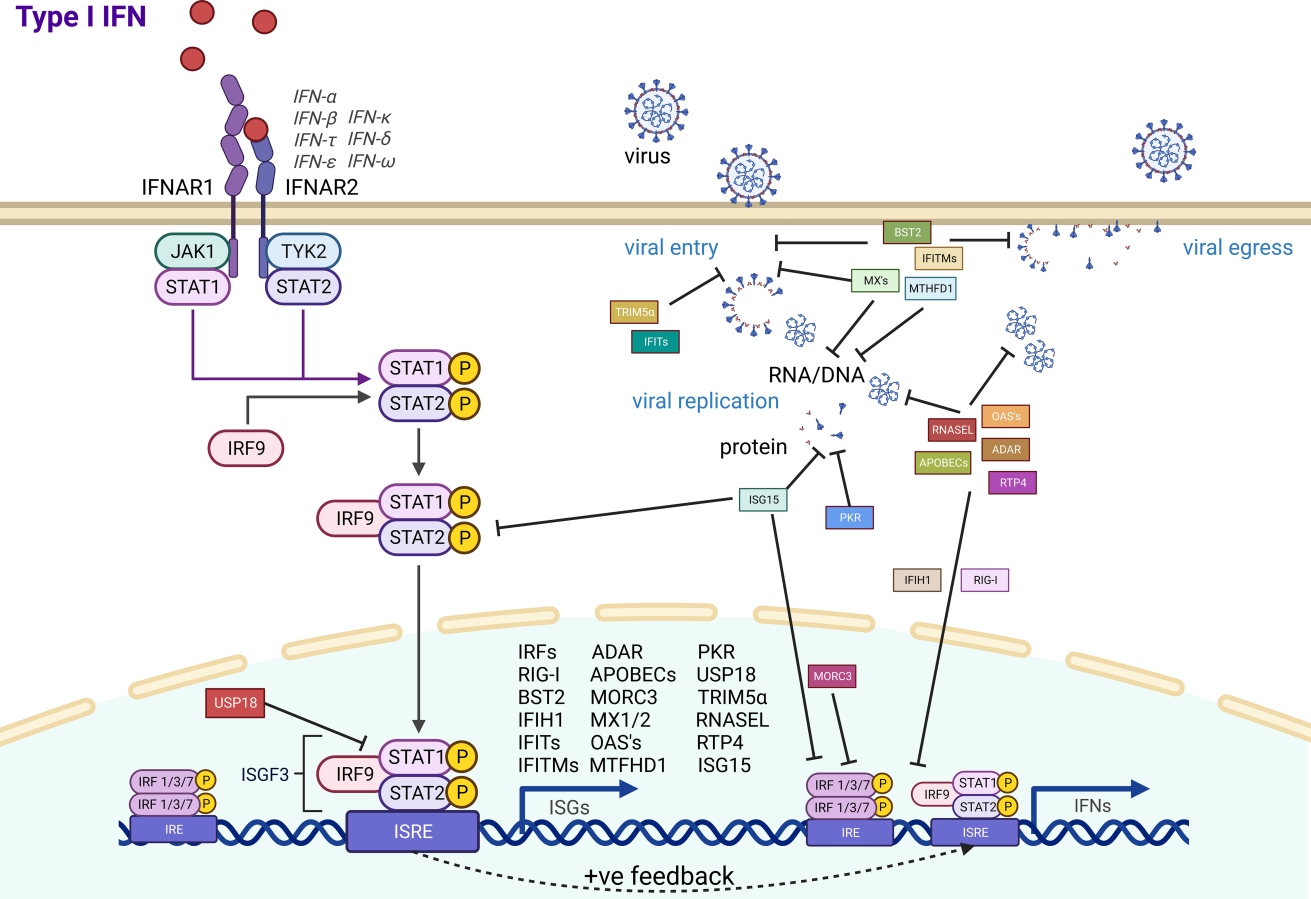
Bat antiviral effector ISG activation and function. Antiviral effector ISGs (square boxes) with experimental evidence in bat cells are indicated with their associated role in inhibiting ISG induction (round boxes) or different steps in the viral life cycle.

The evolution of antiviral effector genes is directly linked to the evolution of the pathogen in an ongoing arms race against our innate immune systems. As bats are associated with being reservoirs of a range of zoonotic viruses, we summarized the antiviral effector ISGs that have experimental evidence for their function (**Figure 1**). A large majority of the immune system is consistently conserved throughout all mammals, with a range of ISGs having antiviral effector function in bats, just as they do in humans. Yet, bats also have some unique adaptations and appear to exhibit a powerful antiviral immune system. Antiviral effector ISGs in bats target almost all steps of the viral life cycle, including entry, uncoating, genome replication, virion assembly, and release. Typical ISGs such as OASs, MX1, and ADAR1 have been identified, and some functional characterization highlights a mostly conserved role in antiviral immunity. Yet, certain branches of the phylogenetic tree and individual bat species appear to have expansion of ISG gene families that may be related to exposure to specific pathogens. Gene expansions of BST2, APOBECs, and PKR, among others, highlight some unique antiviral advantages. The IFN inducibility of other proteins such as MORC3 and RNASEL also provides bats with an advantage over their human counterparts. Moreover, subtle differences in species specificity for subtypes of influenza A virus by Mx proteins suggest that more detailed investigations are needed at the independent species level for bats. With over 1,462 species of bats, there are undoubtedly evolutionary differences to be observed across the order Chiroptera. Some of these uniquely evolved ISG effectors may provide further insight into how we can regulate infection in humans. Potential bat-specific functions from ISGs, IFNs, and IRF transcription factors suggest a unique regulation of innate immunity for a holistic control of virus infection. Overall, the heightened levels of ISGs in the basal state of bats, coupled with differentially evolved functions, may play a key role in inhibiting viral infections. Identifying these key differences between bat and human ISGs allows us to better modulate the function of human ISGs to enhance antiviral immunity.

## Author contributions

AI conceived the idea, DZ and AI jointly wrote the manuscript, edited and reviewed. All authors contributed to the article and approved the submitted version.
